# Patient Educational Needs of Patients Undergoing Surgery for Lung Cancer

**DOI:** 10.1007/s13187-014-0658-2

**Published:** 2014-04-23

**Authors:** Judy King, Paul Chamberland, Anissa Rawji, Amanda Ager, Renée Léger, Robin Michaels, Renée Poitras, Deborah Skelton, Michelle Warren

**Affiliations:** 1 Faculty of Health Sciences, School of Rehabilitation Sciences, Physiotherapy, University of Ottawa, 451 Smyth Road, Ottawa, ON K1N 6 N5 Canada; 2 The Ottawa Hospital, 501 Smyth Road, Ottawa, ON K1H 8 L6 Canada

**Keywords:** Patient education, Lung cancer, Needs assessment, Interprofessional, Physiotherapy

## Abstract

There often exists a discrepancy between the information health care professionals (HCPs) provide to patients in preoperative teaching sessions and the information patients perceive as important. This study’s purpose was to determine what information patients undergoing a lung cancer surgical resection wanted to learn before and after their surgery and also to uncover the information HCPs currently provide to these patients. Ten patients were interviewed preoperatively and postoperatively, and eleven HCPs involved in both their preoperative and postoperative care were interviewed. Emerging themes were noted. Patients reported that the most helpful aspects of the preoperative education included surgical details and the importance of physiotherapy, including exercises. Postoperatively, patients wished they had known more about postoperative pain. HCPs provided information that they felt prepared, informed and empowered their patients. Overall, patients expressed satisfaction with the information they received; they felt prepared for their surgery but not for postoperative pain control.

In 2008, there were 12.7 million people diagnosed worldwide with cancer and 7.6 million deaths associated with cancer [1]. Of all cancers, lung cancer is not only the most commonly diagnosed in the world with 1.61 million new cases per year which accounts for 12.7 % of all new cancers but also the most deadly with 1.38 million deaths (representing 18.2 % of all cancer-related deaths) [1]. The most common treatments for lung cancer include chemotherapy, radiation and surgical resection.

For patients undergoing any surgery, including lung surgery, patient education is seen as critical to their recovery. Education is known to be an essential tool to provide patients with information concerning their health condition, their treatment and their recovery [2]. Patients who receive structured patient education, such as patient education classes, are more likely to follow through with their therapeutic regiment compared with patients who receive unstructured education [3]. Certainly, health care professionals (HCPs) are aware of the importance of providing effective information and advice for patients undergoing surgery [4].

Education material can be presented prior to surgery, during the hospital stay and/or after surgery, and post-discharge. Studies show that preoperative teaching has a beneficial effect on postoperative outcomes for patients undergoing thoracic surgery [5, 6]. Specifically, preoperative anaesthesia teaching has been shown to increase patients’ understanding of options for anaesthesia care, decrease anxiety and fear and decrease analgesic requirements, postoperatively [7, 8]. Preoperative physiotherapy teaching has been shown to decrease complications and even shorten hospital stays [9, 10].

Preoperative patient education can be provided by HCPs in a group setting or in individual teaching sessions. Both settings have their advantages as in groups; patients can decrease their anxiety and gain reassurance while discussing with others having similar conditions, but individual teaching allows for patients to obtain specific information more pertinent to them [11, 12]. Whyte and Grant [13] advanced that providing preoperative education in a coordinated format allows for patients to receive the same information prior to surgery with the overall goal to have the patient become more knowledgeable about their condition and ultimately become a member of the decision making team.

We know from adult learning principles that the amount of information retained from various sources differs across patients [14, 15]. Therefore, patient education needs to be provided using a variety of formats [16–17]. As well, due to other factors that affect patients’ learning, including the stress of having cancer, information needs to be reinforced at different points along the clinical journey (preoperative, postoperative stay), multiple times, and by different HCPs [10, 13].

Yet, despite this knowledge, there is often a disconnection between what HCPs think patients need to know and what the patients want to know [18]. Van Weert [19] demonstrated this discrepancy in patient education for those undergoing cardiac surgery. A key area of discord was around the issue of emotional support. HCPs provided mainly medical information and patients were seldom asked about their fears, anxiety level, understanding of the information or expectations. Furthermore, little reassurance or emotional support was provided.

To further understand and uncover the issues surrounding patient education for patients having lung surgery due to lung cancer, this study was undertaken. The specific purpose of this study was to understand what information patients who were about to undergo lung surgery wanted to learn before and after their surgery and to establish what information is currently provided to these patients by HCPs.

## Methods

### Research Site

The study was conducted at a cancer assessment clinic (CAC) that uses structured preoperative group and individual education sessions to prepare patients for their upcoming surgery and cancer treatments. The interprofessional bilingual (French and English) CAC provides care for a diverse population within a large geographic area. When a person is diagnosed with a lung cancer that can potentially be surgically resected, they are referred to the CAC where they receive the required evaluation and tests for surgery. They also receive a group teaching session that includes a tour of the hospital by a volunteer and a formal presentation by nurse educators. This presentation also includes two short sections presented by the social worker and the physiotherapist. Afterwards, each patient meets one-on-one with the social worker, the physiotherapist and finally the anaesthetist to receive more individualized surgical preparation information.

### Methodological Approach

The study was approved by the Ottawa Hospital Research Ethics Board and the University of Ottawa Research Ethics Board. This qualitative study used a generalized qualitative approach and employed semi-structured interviews to gather data [20]. Ten patients were interviewed preoperatively and postoperatively to obtain insight into their patient education needs, and 11 HCPs were interviewed to uncover their education practices and their self-perceived role in patient education. Qualitative analysis of the interviews was completed in order to extrapolate common themes.

### Participants

Patient participants were recruited in person at the CAC by a bilingual research assistant. Inclusion criteria included being over 18 years of age, either English or French speaking, scheduled to have lung surgery due to lung cancer at the hospital and willing to discuss their patient education needs. Exclusion criteria included plans to have a surgery for a reason other than lung cancer.

HCP participants’ inclusion criteria included being over 18 years of age, either English or French speaking, providing patient education to patients awaiting lung surgery and willing to discuss the patient education they provided. HCPs were excluded if they did not provide patient education.

### Data Collection

To reduce patient participants’ time at the hospital, they had the option of completing the preoperative interview in person after their CAC session or by telephone at a later time the same day. All postoperative interviews were conducted over the telephone. Both interviews were guided by the same questionnaire that included semi-structured and close-ended questions. During the postoperative interview, patients were reminded of their preoperative comments. HCPs’ interviews were conducted in person and also included both close-ended and semi-structured questions.

### Data Analysis

Interviews were analysed using a qualitative naturalistic inquiry approach [21]. Inductive analysis of the data allowed for the identification of themes. Each of the patient’s and HCP’s interviews was read individually to determine a narrative response to each of the major questions asked. For the patient participants, there was a focus on the information they had found most helpful as well as information they had found unclear or missing. As well, the interviews of each of the participants were read to determine themes emerging across the interviews. For the HCPs, a descriptive narrative was developed of all the information and resources that they used in teaching patients.

### Findings

#### Health Care Professionals Results

##### Demographics

Of the 11 HCPs recruited for the study, there were two registered nurses (RN), one physiotherapist (PT), two social workers (SW), two physicians and four thoracic surgeons. Five of the HCPs interviewed had been with the CAC since its inception in 2006, and ten of the 11 HCPs had more than 3 years of experience working with patients diagnosed with lung cancer.

##### Topics Discussed in Education Sessions

A variety of topics were discussed with patients during education sessions. The topics covered by each HCP depended on their profession but were also specific to the patient’s phase of recovery (i.e. before or after surgery, during hospital stay or after discharge). Preoperatively, the HCPs repeated similar educational information addressing the topics of, namely, medications, pain control, exercises, preoperative procedures, common complications, role of the HCPs, role of the patient, role of family members, discharge planning and follow-up. Other issues covered preoperatively included diet, postoperative procedures and home care services. Postoperatively during the hospital stay, fewer HCPs provided information for patients; here, the topics included pain control and pain medication, the role of the specific HCP and patient follow-up after discharge. There were other issues discussed which were similar to those presented preoperatively. Following the patient’s discharge from the hospital, the patients would attend a follow-up visit at the CAC. At this follow-up visit, they would receive information about further treatment they may require.

##### Format and Resources

While all HCPs used verbal communication to transmit information, they also used written materials to supplement their verbal communication; the aim of using both methods was to support patients’ understanding of the material. Written information included the Canadian Cancer Society’s resources, a specific exercise booklet (*Post-Op Thoracotomy Exercise*), and a printout of the Power Point presentation used in the group education session. Some of these resources had pictures and drawings while others did not. The most commonly used booklet was *Lung Cancer: Information Guide and Personal Record*, a compilation of lung cancer information that covers such topics as diagnosis, personal thoughts and coping strategies. Additional materials included the admission booklet from the hospital, S*ternotomy Restrictions* (a hospital produced activity guide), a chemotherapy and radiation booklet and the pre-admission unit booklet for medication information. The social workers also provided patients with logistical details regarding options for convalescent care postoperatively as well as information about parking and finances.

##### HCP’s Perception of their Role

Each of the HCPs discussed the primary goals of their education sessions. Three common themes surfaced as follows: HCPs all wanted to adequately prepare patients for their surgery and recovery, make sure patients understood the HCPs unique role and responsibilities, and support and reassure patients about the surgery. These findings could be categorized as HCPs wanting to prepare, inform and empower their patients.

#### Patient Participants Results

##### Demographics

Ten patient participants, five men and five women, were recruited from four preoperative group education sessions at the CAC. Their ages varied between 50 and 79 years old. Seven patient participants primarily spoke English, three spoke French and either Vietnamese or Italian at home. Eight patient participants lived with a spouse and two lived alone. Six patient participants required more than 40 min to drive to the CAC, while the rest lived between 10 to 40 min from the hospital. Nine patient participants were retired, and only one patient continued to work fulltime. Hospital length of stay varied from 1 to more than 7 days. Postoperatively, the interviews were conducted after the patient had been home for at least 5 days. This time was chosen to allow enough time for the patient to get home and into some sort of routine, but not so much time that they might forget details of their experience. Three patient participants were receiving home care services at the time of the postoperative interview.

##### Topics Discussed

When patient participants were asked to discuss the most helpful and most surprising information, as well as what was unclear in the education presentation preoperatively, there were no common themes that emerged. Although all patient participants found the information to be very helpful, they reported that the information about the surgery itself and the session with the physiotherapist where they learned about breathing exercises were most helpful. When asked about the most surprising information, each participant’s answer was unique and ranged from the importance of physiotherapy interventions including exercises, to details about the surgery, information about the functioning of the lungs and how a cancer in the lung can originate elsewhere. In general, participants felt there was no unclear information and one participant noted that: “if it was unclear, you got to ask on the spot and they answered your questions and explained it to you”. When asked about what information was the most helpful, each participant responded with the same type of information they found most helpful preoperatively. All the participants mentioned that they still were keeping up with the exercises prescribed by the physiotherapist. All participants confirmed that the information was clear. Two participants wished that they had received their preoperative test results and the surgery plan prior to surgery. The most notable difference between preoperative and postoperative comments was postoperative comments regarding a wish to know more about the level and timing of pain associated with surgery prior to the surgery. This appears to be a considerable need not met. One participant expressed the need to know “how long things are going on after surgery, like pain. How long will I have pain?”

##### Format and Resources

In terms of teaching format, the majority of patient participants stated they liked receiving information directly from a HCP and reading on their own both preoperatively and postoperatively. Patient participants commented that they found the *Pulmonary Resection Booklet* and *Post-Thoracotomy Exercise Booklet* the most helpful of all the print material given to them preoperatively. Looking back at their journey, patients reflected that the booklet *Post-Thoracotomy Exercise Booklet* and the *Lung Cancer: Information Guide and Personal Record* were helpful in answering their questions.

##### Overall Experience

The CAC education session was an overall positive experience for the participants in the face of being diagnosed with cancer. When asked to give feedback on their confidence and satisfaction, all patients participants felt very satisfied with the information provided and very prepared for their surgery. From their experiences, participants provided a number of suggestions to improve the experience for future patients. These suggestions included allowing for a break in the long day, having a doctor present for the education session and simplifying some of the language while adding pictures to the presentation to improve their understanding of the material.

## Discussion

An interprofessional team approach to preoperative patient education allows for various topics to be presented to patients by different members of the team. These topics are profession specific. Studies have found that to enhance information retention, topics need to be discussed on several occasions, by more than one HCP [10, 12, 13]. This may have been supported in this study by the fact that two of the topics presented both preoperatively and postoperatively, regarding surgery and importance of physiotherapy, were perceived as the most helpful information presented at both time periods. It is important to note that although repetition is likely to enhance retention, there is a limit to the amount of new information patients will remember [22]. Repeated information should be carefully selected. As well, all HCPs who present a topic to the patient should be aware of what other members of the team are saying about it, to ensure patients receive consistent messages.

Pain and pain management were topics the majority of HCPs discussed preoperatively, yet preoperatively, patients did not perceive this as one of the most helpful pieces of information. Following their preoperative education sessions, patients did not mention that this information was unclear. However, after returning home postoperatively, patients wished they had been better informed regarding pain before their surgery. Although the topic was addressed repeatedly in the preoperative and again postoperative phase by HCPs, the fact that patients perceived they lacked information regarding pain may be as a result of how HCPs described the pain to patients. Perhaps, pain simply needs to be addressed further or differently preoperatively and postoperatively. As well, it may be the case that patients are more concerned with surviving the surgery preoperatively than concerned with their postoperative recovery. This is noted from the HCPs who commented that the questions that patients ask them are around cure and if they can “get the cancer out”, with the surgery.

When meeting with patients and families, HCPs provided or referred to various resources in order to promote a better understanding of what was taught. The amount of information can be overwhelming and retention may be suboptimal as patients become saturated with information. One patient participant noted that

“I think there is too much information in the green leaf book (*Lung Cancer: Information Guide and Personal Record*). It’s a difficult operation and I don’t want to remember some things”.

Multiple patient education methods are needed to transfer knowledge, including information provided in a written format, the use of drawings and the repetition of information to facilitate retention [13]. Therefore, as most of the HCPs did prior to surgery, it is imperative to provide written material and visual aids for patients to consult after the education sessions. But, this information needs to be designed using plain language and clear design so that it is accessible to people who might be living with limited literacy [23]. The gap between the literacy demands of the system and the literacy abilities of patients must be narrowed to improve both the effectiveness of the health care system and the quality of life for the patients [24]. In terms of learning formats, preoperatively, the most popular method to receive information was directly from a HCP, while after their surgery, patients preferred reading independently. This supports the idea that adult learners need different methods to meet their needs.

A few of the patients both preoperatively as well as postoperatively noted that they or their family members seek information from the internet. Peterson and Fretz [25] have reported an increased use of the internet by patients to obtain information pertinent to their cancers. Certainly, patients, as part of their self-directed learning, are using the internet as a source of health information about their condition [26].

Perhaps the age range of the patients could explain the limited use of the internet in this study; these patients may not have known how to search for additional information. Alternatively, our patient participants may not have wanted to learn more. At least preoperatively, they may have been finding their cancer diagnosis and planned surgery overwhelming; the thought of additional information was too stressful. In fact, one participant noted, “Friends looked for me and found things on the internet but they thought it was too scary so they didn`t give it to me”.

Lilja [27] found that patients who had received detailed information preoperatively had higher levels of stress postoperatively than the group who had received less information. The lack of attempts by the participants to find external resources of information may also reflect that patients at that stage are primarily concerned with survival versus recovery.

## Conclusions

For a patient education session to be efficient and achieve the goals of decreasing anxiety, empowering through knowledge and improving postoperative outcomes, information provided has to be perceived by the patient as relevant. Good communication is required between the HCPs to cover all required topics and only repeat those of utmost importance. The information needs to be shared with patients at various phases or the continuum of care using different formats to maximize learning. In the case of this study, patients reported satisfaction with their preoperative education when they reflected on this both preoperatively and postoperatively. Furthermore, all patients felt very prepared before their surgery and when looking back once the surgery had been done, five out of six still felt very prepared while one now felt he had been moderately prepared.

It is hoped that the results of this study will help inform HCPs of the education needs of patients who are undergoing lung surgery so that they may better tailor education sessions to meet patient needs and expectations. Although a limitation of this study was a small sample size and there was not a uniformity in the patient’s length of hospital stay and post-op management. These varied experiences did not seem to impact the perceptions of the participants. Based on the education needs expressed by the participants in this study, pain management is definitely a topic that needs to be addressed more fully both preoperatively and postoperatively. Our next step in this research program will be to further examine the topic of pain including how the issue is discussed by HCPs with patients including the words used to describe the pain experience by both HCPs and patients. Furthermore, we look forward to comparing the current data gathered from an Ottawa clinic to data from across Canada.
